# Comparison of Hand Dexterity According to Selected Thermal and Auditory Stimuli

**DOI:** 10.3390/ijerph20010765

**Published:** 2022-12-31

**Authors:** Hee-Soon Woo, Chiang-Soon Song

**Affiliations:** 1 Department of Occupational Therapy, School of Medicine, Wonkwang University, 460 Iksandae-ro, Iksan, Jeonbuk 54538, Republic of Korea; 2 Department of Occupational Therapy, College of Natural Science and Public Health and Safety, Chosun University, Chosundae-5gil, Dong-gu, Gwangju 61452, Republic of Korea

**Keywords:** thermal stimuli, auditory stimuli, sensory stimuli, hand dexterity, grooved pegboard test, Purdue pegboard test

## Abstract

The hand plays a crucial role in our daily lives and affects the quality of life. Sensory stimuli can affect the activation of the autonomic nervous system to control body homeostasis and finger motions. This study aimed to determine the optimal thermal and auditory stimuli that affect hand dexterity. The experiment included thirty healthy adults. In the experiment, the thermal stimuli were classified into 20, 30, and 40 °C. The auditory stimuli were classified into fast- and slow-tempo music. Each stimulus was randomly provided for 5 min and hand dexterity was tested with the Groove and Purdue pegboard tests. After each stimulus and test was conducted, a resting time of 20 min was provided before the next stimulus. When the thermal stimulus of 30 °C and auditory stimulus of fast-tempo music was provided, the completion duration of the hand dexterity test was the shortest. Except the thermal stimulus of 20 °C, all thermal and auditory stimuli induced increased hand dexterity, compared to the non-stimulated condition. Among the five categories of thermal and auditory stimuli, the thermal stimulus of 30 °C and auditory stimulus of fast-tempo music were the most effective in improving hand dexterity.

## 1. Introduction

The hand is one of the most sensitive body parts. It is also the most functional part in our daily lives, on account of its detailed motions [1]. It is an organ that has essential motor functions and activities that facilitate survival, maintenance, and information retrieval to interact with the environment [2]. Jebson et al. [3] and Exner [4] define the hand as an important instrument for play, daily life operations, and other diverse activities. 

The main functions of the hand include gripping, putting and operating, and affect the essential components of daily life, such as adjustment, stability, sensory expression, and sensory delivery [5]. The ability to move the arms and hands with optimal speed is necessary to perform various functions, including the adjustment of things in technical and right directions [6]. This ability is called hand dexterity, and is mainly reflected by the measurement of various functions of the hands [5].

Patients with stroke, spinal cord injuries, or mental disorders and older individuals show lower hand dexterity due to disease or aging. Most stroke patients experience fine motor and sensory control damage, which limits their daily life activities [7]. Patients with neck and spinal cord injuries have complete or incomplete motion or sensory damage, which often results in decreased hand dexterity. In particular, patients with paraplegia require greater hand dexterity because they make significant use of their upper body parts, particularly their hands, to perform daily life activities. Patients with mental disorders also experience operations related to hand functions, such as writing, adjusting, and touching [8]. Treatments that enhance sensory control function and movement ability can improve hand dexterity; these include kinesthetic training, sensory integration therapy, and the cognitive-motor approach. 

Kinesthetic training utilizes the neurophysiological principle to facilitate reactions of the local nerves and muscles. Grip power, one of the focuses of motor and sensory trainings, has a positive influence on the improvement in hand function. Lateral pinch strength, tip pinch strength, and three jaw chuck strength are coordinated through grip power, and the optimal functional performance of the hands in daily life can be ensured by adjusting the grip power [9]. Sensory integration therapy induces adjustment reactions suitable to the external environment through sensory stimuli, and comprehensively affects the motor functions of children with developmental coordination disorder. Studies on children with developmental coordination disorder have focused on tasks such as writing and posture adjustment [10,11].

Among the therapeutic approaches described above, the Rood approach is based on neurophysiological principles. It operates on the understanding that sensory stimuli cause motor reactions, and these sensory stimuli prompt or restrict muscle tension [2]. Sensation is a cognitive phenomenon that occurs when external stimuli are received. It is integrated and processed through a higher center and prevents or minimizes potential damage to the body; therefore, it is closely related to motion [12,13]. In particular, the initial bodily sensations are stored in the brain, used to create effective movement reactions, and are necessary to perform fine movements [14].

Sensory stimuli that affect the body generally include the tactile, auditory, and olfactory stimuli. These senses stimulate the autonomic nerves to adjust bodily balance and generate effective motor reactions [14,15]. Among the tactile stimuli, thermal therapy prompts a balance between the sympathetic and parasympathetic nervous systems and facilitates homeostasis of the autonomic nervous system, restricting frequent activation of the sympathetic nervous system and simultaneously activating the parasympathetic nervous system [16]. With respect to auditory stimuli, the tempo affects the activation of both the sympathetic and parasympathetic nervous systems, causing physiological reactions [17]. As a result, various sensory stimuli are thought to affect the facilitation and activation of the autonomic nervous system, maintaining the homeostasis of the human body, and affecting motion. Although all sensory stimuli do not prompt movement, they affect both the facilitation and restriction of movement depending on the degree of stimulation. 

According to Clark [18], manual dexterity is limited at a temperature of 13 ℃, whereas, at 16 °C, no influence is observed. Gaydos and Dusek [19] reported that finger dexterity was significantly reduced at 10–13 °C. Lee et al. [20] reported that a temperature of 20 °C affects the activation of hand function. Rhee et al. [21] reported that a temperature of 38–42 °C is helpful in activating hand function and enhancing concentration. Shim [22] reported that the autonomic nervous system affects the degree of activation according to the music speed and affects both concentration and hand dexterity. Hong and Kim [23] reported that auditory stimuli enhance concentration and influence fine motor control. As a result, sensory stimuli can affect autonomic nervous system activation to control homeostasis and finger motions. 

In previous studies, among various sensory stimuli, auditory and thermal stimuli had a positive influence on increasing hand dexterity. However, depending on the degree of a given stimulus, there may be changes in hand dexterity. Therefore, this study aims to test the degree to which thermal and auditory stimuli affect manual dexterity, with a focus on the degree of stimulus. We believe that application of both thermal and auditory stimuli in therapy for patients with low hand dexterity can provide better results. 

## 2. Materials and Methods

### 2.1. Subjects 

The experiment was conducted with 30 healthy adults. The inclusion criteria comprised people without a medical history of musculoskeletal and neurological diseases and with no difficulty in operating the joints in their upper bodies. In order to exclude the variables that affect mobility by sensory input as much as possible, individuals who did not regularly exercise were included. As a result, 10 male and 20 female adults participated in the experiment. All participants provided signed informed consent for participation in the study. 

### 2.2. Experiment Design 

The experiment duration was 13–20 November 2021, for a total of 8 days. The experiment was conducted for 5 of the 8 days. A total of five stimuli, including three thermal stimuli at 20, 30, and 40 °C, respectively, and two auditory stimuli of fast and slow tempo music, were provided to the participants. For each stimulus, manual dexterity was evaluated using an evaluation instrument. Subsequently, to offset the learned effects of the evaluation, the order of the stimuli was chosen by the participant and applied randomly. The room temperature was set at 22–24 °C with a humidity of 40%. To control the environment, the experiment was conducted in as quiet an environment as possible. 

### 2.3. Process

**Figure 1:**
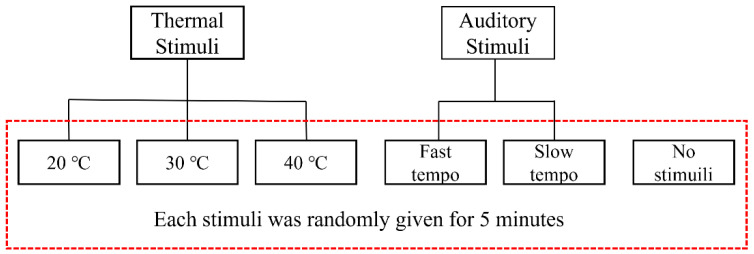
Process of the experiment.

In this study, 3 types of thermal stimulation and 2 types of auditory stimulation were provided to the subjects. In addition, a total of six tests, including performance at normal room temperature (22–24 ℃) without auditory stimulation, were conducted for use as a baseline. Hand dexterity evaluation was conducted within 3 min after the stimulus was provided, and each test was randomly assigned to each subject to eliminate the learning effect caused by repeated use of evaluation tools (Figure 1).

### 2.4. Input of Sensory Stimuli 

#### 2.4.1. Input of Thermal Stimuli 

Lee et al. [20] reported that concentration increased at 20 °C; additionally, Rhee et al. [21] reported that manual dexterity increased at 40 °C. In this study, we evaluated hand dexterity at both these temperatures and at 30 °C, the mean of both. The participants were subjected to thermal stimuli by placing their hands in water at 20 °C, 30 °C, and 40 °C for 5 min. After 5 min of thermal stimuli and before conducting the evaluation, the water on the hands was removed by a towel in order to prevent the peg from slipping. In this case, to avoid providing tactile stimuli to the hands, a smooth towel was used. For maintenance of the provided temperature stimulus, the evaluation was performed immediately within 3 min after the stimulus was provided.

#### 2.4.2. Input of Auditory Stimuli 

In Shim’s study [22], four musical numbers were used to increase concentration, whereas Hong and Kim [23] used 12 numbers. Among the 16 music numbers used in the two studies, fast-tempo (Thunder and Lightning by Johan Strauss) and slow-tempo (Tristesse by Chopin) music were chosen and provided. Specifically, music with a fast tempo was provided with an average of 120 beats per minute (BPM) or more, and music with a slow tempo was provided with an average of 40 to 60 BPM. Auditory stimuli were also provided for 5 min to maintain consistency with the thermal stimuli. In order to avoid interference from other stimuli, the participants were asked to listen to the music with their eyes closed. Music was provided through speakers placed on the left and right in front of the subject and 5 m away, and was provided for 5 min at from 80 to 90 decibels corresponding to a moderate volume. For retention of the presented auditory stimulus, evaluation was performed within 3 min after stimulus presentation.

### 2.5. Evaluating Instrument 

#### 2.5.1. Grooved Pegboard

To evaluate the dexterity of both the dominant and non-dominant hands according to the sensory stimuli, a grooved pegboard consisting of 25 pegs on each hole was used for the evaluation of neurological tests, vision-motor coordination, and work tests. The test of this evaluation instrument with five lines was conducted after application on the dominant hand, followed by the non-dominant hand. In the case of the dominant hand, the peg was inserted from the non-dominant to the dominant hand. In the case of the non-dominant hand, the peg was inserted from the dominant to the non-dominant hand. When conducting the test, the upper lines were filled followed by the lower lines. The time from the ringing of the ‘start’ bell until filling of the final peg was measured. The pegboard was fixed to the desk to avoid its movement. The credibility between the test and retest for this instrument was r = 0.75 for the dominant hand and r = 0.74 for the non-dominant hand, respectively [24].

#### 2.5.2. Purdue Pegboard

The Purdue pegboard for testing hand dexterity according to sensory stimuli was developed by Tiffin, an industrial psychologist [25]. The instrument consists of a small peg operated in a hall and measures the accuracy and speed of the hand by operating, gripping, and placing pegs in the holes The test can measure hand dexterity by counting the number of pins with the dominant hand and the non-dominant hand for 30 s, and can evaluate the ability to assemble using both hands with a pin, washer, and caller for 60 s. The credibility between the test and retest was r = 0.60–0.79 [25]. In this study, only the evaluation of assembly ability using both hands was applied to the Purdue pegboard. The original Purdue pegboard evaluation was to score the number of assembled parts for 60 s, but in this study, the time to create 8 sets of assemblies using 4 parts was measured and used for the results to compare the execution time.

### 2.6. Statistical Analysis

To compare the degrees of both thermal and auditory stimuli on manual dexterity, the measured data were classified into data for the dominant, the non-dominant, and both hands. With an evaluation using both the grooved pegboard and Purdue pegboard, the measured data were collected. The time spent on both the evaluation instruments was measured, and a shorter time meant higher hand dexterity. The collected data were analyzed using SPSS Version 22.0. Normality was confirmed in the dominant hand, non-dominant hand, and both hands through the Shapiro–Wilk test. To compare the results of thermal stimuli, one way repeated ANOVA was performed to confirm the effect of each stimulus, and Bonferroni Post-Hoc was applied for comparison between stimuli. For auditory stimuli, a paired-sample *t*-test was used as a Post-Hoc test.

### 2.7. Ethical Approval and Consent to Participate

All the procedures performed in this study were in accordance with the ethical standards of the institutional and/or national research committees and the 1964 Helsinki declaration and its later amendments or comparable ethical standards. All the participants provided informed consent to participate in the study. The protocol was approved by the ethics committee of the Institutional Review Board of Wonkwang University (WKIRB-202201-HR-002).

## 3. Results

### 3.1. Differences in Hand Dexterity According to Thermal Stimuli

An analysis of hand dexterity results according to varying thermal stimuli revealed that the dominant hand showed the best result at 40 °C, while the non-dominant hand and assembly with both hands showed the best result at 30 °C (Table 1 and Table 2). Analysis of the integrated results for dominant, non-dominant, and assembly with both hands at 30 °C revealed a shorter duration of time by an average of 3.59 s than that without stimuli. As seen in Table 2, at 20 °C, the comparisons at 30 and 40 °C were statistically significant (*p* < 0.05). In the comparison between 30 and 40 °C, the results were not statistically significant (*p* > 0.05).

### 3.2. Differences in Hand Dexterity According to Auditory Stimuli

In all the variables of the hand dexterity test according to the auditory stimuli, there were statistically significant differences (Table 3 and Table 4). When comparing the evaluation time for all three hand conditions, the average of the three was shortest with fast-tempo music (Table 3). With fast-tempo music stimuli, the dominant hand, the non-dominant hand, and assembly with both hands were observed to be 2.94 s, 3.9 s, and 5.61 s faster in completing the evaluations. 

As seen in Table 4, when comparing the average time for hand dexterity in the dominant, the non-dominant, and assembly with both hands according to the kinds of stimuli, the differences in the results between fast- and slow-tempo music were statistically significant (*p* < 0.05). Therefore, hand dexterity improved when auditory stimuli were provided. Upon providing a fast tempo, the time was 4.15 s shorter, while for slow tempo, the time was 2.48 s shorter, with the results showing that hand dexterity improved more with fast-tempo music. 

## 4. Discussion

Hand dexterity, the ability to move the hands and arms quickly in order to adjust things in technical and right ways, functions as an important factor in our daily lives [5]. In previous studies on hand dexterity, thermal and auditory stimuli were helpful in improving hand dexterity; however, precise levels of stimuli required were not suggested. Therefore, this study sought to determine whether thermal or auditory stimuli were more effective when both types of stimuli are provided to ordinary individuals. It supplemented the limitations of the previous studies, classified thermal and auditory stimuli in detail, and determined the level that was most effective for hand dexterity.

In the case of thermal stimuli, Lee et al. [20] reported that the frequency of the brain waves concentrating at 20 °C increased and affected the activation of hand functions. Rhee et al. [21] reported that a temperature of 38–42 °C induced concentration and helped to activate hand functions. Additionally, Daanen [26] reported that upper dexterity and grip power increased as the temperature of the hands increased when the temperature changed in the ranges of 6–32 °C. Our results showed that hand dexterity improved in the order of 30 °C, 40 °C, and 20 °C. This was not consistent with the previous results, which showed that dexterity increased as the temperature increased, although the hand dexterity increased up to 32 °C. At 40 °C, hand dexterity was lower. It was observed that as the time interval was not large, the results differed on an individual basis. 

In the case of the dominant hand, a lower level of performance was shown at 20 °C compared to when there was no stimulation. This is a different result from Lee et al. [20], which reported enhancement of hand function through EEG activation at 20 °C. The authors speculated that this decrease in function was due to a lower temperature than the room temperature of 24 °C, which was used as a baseline, but the results of the non-dominant hand and the assembly using both hands showed the opposite result. There have been no studies to date showing that stimulation of the autonomic nervous system through external stimuli responds differently to the dominant side and the non-dominant side. A more in-depth follow-up study on the cause of the difference between the dominant hand and the non-dominant hand seems to be needed, even at the same temperature.

In the case of auditory stimuli, Shim [22] and Hong and Kim [23] used 16 alpha wave music, known to affect hand function by improving concentration. This signified that auditory stimuli led to improved concentration and increased manual dexterity. In this study, a fast-tempo auditory stimulus was more helpful for improving hand dexterity than slow-tempo stimuli. In addition, compared to slow-tempo music, fast-tempo music showed better effects in improving hand dexterity, as demonstrated by Kim [17], who showed that fast-tempo music led to physiological reactions. 

This study attempted to determine the degree of improvement in manual dexterity. In previous studies, as the thermal sense was delivered to a higher center, the speed of the blood flow in the muscles and brain increased. As a result, more heat was generated in the muscles, facilitating activation and improved performance. The delivery of heat to the muscles has been used as the main mechanism for enhancing muscle functions and preventing injuries. Auditory stimuli using alpha wave music activated the autonomic nervous system, and the stimuli were delivered to the brain, with psychological, physical, and affective influences. As a result, both thermal and auditory stimuli can function as a medium to lead to additional physical ability in dexterity than what an individual possesses. 

Accordingly, when treating individuals who need to improve their lowered hand functions, such as stroke patients or those with spinal cord injury or mental disorders, if such a medium is used properly, it will have a significant impact during a short therapy period. The sensory stimuli used in this study are easily handled and do not require special techniques, and they can be easily applied in the clinical field. 

This study has several limitations. First, it was conducted on healthy individuals and the deduced results may be insufficient to be applied to patients, and there may be differences between the stimuli and effects on an individual basis. Second, in this study, classical music used in previous studies was applied for auditory stimulation, but in the case of music, each person’s taste and preference is different, so it is judged that the variables related to this also affected the results. Third, evidence regarding the interaction between the senses and motions remains lacking; moreover, it is expected that additional studies on this topic will have to be conducted. In the near future, this method may be applied to patients who need therapy to improve manual functions, and the therapeutic evidence can be subsequently examined. 

## 5. Conclusions

The purpose of this study was to review the degree of stimuli that are most effective for hand dexterity by dividing the thermal and auditory stimuli in individuals with lower manual dexterity. Hand dexterity was tested on 30 healthy adults, and thermal stimuli of 20 °C, 30 °C, and 40 °C and auditory stimuli of slow- and fast-tempo music were used. The results showed that, for auditory stimuli, the manual dexterity test time was shortest for fast-tempo music. In addition, except at 20 °C, the application of both thermal and auditory stimuli resulted in increased manual dexterity compared to testing without stimuli. However, there was no correlation between the thermal and auditory stimuli. This means that different stimuli, both thermal and auditory stimuli, cannot be compared with identical criteria. Therefore, it is unknown which is more effective between thermal stimulus at 30 °C and auditory stimulus with fast-tempo music. However, in this study, among the classified thermal and auditory stimuli, stimuli at 30 °C and with fast-tempo music were the most effective. 

The findings may have been affected by environmental influences on the test laboratories, differences between testers, and difficulties in temperature maintenance. Therefore, in future studies, experiments should be conducted with consistent temperature stimuli in a controlled environment, excluding differences between testers; furthermore, the preparation of therapy rooms may create a convenient way to provide such an intervention. 

## Figures and Tables

**Table 1:** Changes in hand dexterity according to the thermal stimuli.

	Dominant Hand (Grooved Pegboard)		Non- Dominant Hand (Grooved Pegboard)		Assembly with Both Hands (Purdue Pegboard)		Total	
	Mean ± SD (sec.)	Variance	Mean ± SD (sec.)	Variance	Mean ± SD (sec.)	Variance	Mean ± SD (sec.)	Variance
Non-Stimuli	62.08 ± 6.25		67.09 ± 8.54		55.91 ± 10.52		61.70 ± 10.55	
20 °C	62.98 ± 7.47	+0.9	65.71 ± 8.91	−1.38	54.45 ± 10.29	−1.46	61.05 ± 10.09	−0.65
30 °C	60.76 ± 6.74	−1.32	63.83 ± 8.21	−3.26	49.73 ± 10.80	−6.18	58.11 ± 10.58	−3.59
40 °C	60.66 ± 7.26	−1.42	64.48 ± 8.40	−2.61	50.67 ± 9.91	−5.24	58.60 ± 10.31	−3.10

**Table 2:** Comparison according to the different types of thermal stimuli.

Thermal Stimulus (I)	Thermal Stimulus (J)	Difference of Average (I-J)	Level of Significance (*p*)
20 °C	30 °C	2.938	0.001
	40 °C	2.444	0.012
30 °C	20 °C	−2.938	0.001
	40 °C	−0.493	1.000
40 °C	20 °C	−2.444	0.012
	30 °C	0.493	1.000

**Table 3:** Differences in hand dexterity according to auditory stimuli.

	Dominant Hand (Grooved Pegboard)		Non-Dominant Hand (Grooved Pegboard)		Assembly with both Hands (Purdue Pegboard)		Total	
	Mean ± SD (sec.)	Variance	Mean ± SD (sec.)	Variance	Mean ± SD (sec.)	Variance	Mean ± SD (sec.)	Variance
Non-Stimuli	62.08 ± 5.40		67.09 ± 8.52		55.91 ± 10.55		61.70 ± 10.21	
Fast-tempo	59.14 ± 5.59	−2.94	63.19 ± 7.47	−3.9	50.30 ± 10.58	−5.61	57.55 ± 9.70	−4.15
Slow Tempo	61.48 ± 7.22	−0.6	64.29 ± 8.47	−2.8	51.89 ± 10.83	−4.02	59.22 ± 10.35	−2.48

**Table 4:** Comparison according to the types of auditory stimuli.

Auditory Stimulus (I)	Auditory Stimulus (J)	Differences of Averages (I-J)	Level of Significance (*p*)
Fast-tempo	Slow Tempo	−1.673	0.021
Slow Tempo	Fast-tempo	1.673	0.021

## Data Availability

Not applicable.
